# Phylogeny and Metabolic Potential of the Methanotrophic Lineage MO3 in Beijerinckiaceae from the Paddy Soil through Metagenome-Assembled Genome Reconstruction

**DOI:** 10.3390/microorganisms10050955

**Published:** 2022-05-01

**Authors:** Yuanfeng Cai, Juanli Yun, Zhongjun Jia

**Affiliations:** 1 State Key Laboratory of Soil and Sustainable Agriculture, Institute of Soil Science, Chinese Academy of Sciences, Nanjing 210008, China; 2 State Key Laboratory of Microbial Resources, Institute of Microbiology, Chinese Academy of Sciences, Beijing 100101, China; yunjl@im.ac.cn

**Keywords:** methanotroph, glyoxylate shunt, paddy soil, metagenome-assembled genome, *pmoA*

## Abstract

Although the study of aerobic methane-oxidizing bacteria (MOB, methanotrophs) has been carried out for more than a hundred years, there are many uncultivated methanotrophic lineages whose metabolism is largely unknown. Here, we reconstructed a nearly complete genome of a Beijerinckiaceae methanotroph from the enrichment of paddy soil by using nitrogen-free M2 medium. The methanotroph labeled as MO3_YZ.1 had a size of 3.83 Mb, GC content of 65.6%, and 3442 gene-coding regions. Based on phylogeny of *pmoA* gene and genome and the genomic average nucleotide identity, we confirmed its affiliation to the MO3 lineage and a close relationship to *Methylocapsa*. MO3_YZ.1 contained *mxaF*- and *xoxF*-type methanol dehydrogenase. MO3_YZ.1 used the serine cycle to assimilate carbon and regenerated glyoxylate through the glyoxylate shunt as it contained isocitrate lyase and complete tricarboxylic acid cycle-coding genes. The ethylmalonyl-CoA pathway and Calvin–Benson–Bassham cycle were incomplete in MO3_YZ.1. Three acetate utilization enzyme-coding genes were identified, suggesting its potential ability to utilize acetate. The presence of genes for N_2_ fixation, sulfur transformation, and poly-β-hydroxybutyrate synthesis enable its survival in heterogeneous habitats with fluctuating supplies of carbon, nitrogen, and sulfur.

## 1. Introduction

Aerobic methane-oxidizing bacteria or methanotrophs are a distinct group of bacteria that use methane as their main carbon and energy source [1,2]. The currently described aerobic methanotrophs are affiliated to Alphaproteobacteria (also known as type II), Gammaproteobacteria (type I), and Verrucomicrobia. The two methanotrophic families within Alphaproteobacteria are Methylocystaceae and Beijerinckiaceae [3,4,5]. These methanotrophs convert methane to methanol by using methane monooxygenase (MMO), which exists in particulate (pMMO) or soluble (sMMO) forms [2]. The *pmoA* gene encoding the beta-subunit of pMMO is present in all aerobic methanotrophs except *Methylocella*, *Methyloferula*, and a species of *Methyloceanibacter* [6,7,8]. The phylogenetic analysis of *pmoA* gene sequences in the GenBank database shows that about 20 *pmoA* lineages contain cultured representatives, and there are also more than 20 *pmoA* lineages have no cultured representative, such as upland soil cluster alpha (USCα), upland soil cluster gamma (USCγ), Rice Paddy Clusters, and the Lake Washington Clusters [9,10].

Currently, the analysis of metagenome-assembled genomes (MAGs) is an important approach to investigate the metabolism of these uncultivated lineages and some novel methanotrophs [11]. The reconstruction and analysis of a MAG of USCγ (type I) confirmed the presence of a nearly complete serine pathway of type II methanotrophs rather than the ribulose monophosphate (RuMP) pathway that is common in type I methanotrophs [12]. The functional analysis of a MAG of USCα revealed that this lineage may need to grow in biofilms, and this feature may be one of the reasons for their extremely slow recovery from disturbance [13]. A novel methanotrophic member of Hyphomicrobiaceae, which contains no known methanotroph yet, is predicted by the presence of MMO-like coding genes in two Hyphomicrobiaceae MAGs recovered from a fen sample [14]. In addition, MAG analysis can uncover some new features and function details of the well-characterized methanotrophs [15,16]. However, it is important to note that the MAG approach also has some limitations, such as assembly errors and misbinning of fragments from other genomes which may lead to incorrect evolutionary and ecological insights [17,18].

Beijerinckiaceae methanotrophs, also known as type IIb, contains 3 genera (i.e., *Methylocapsa*, *Methylocella*, and *Methyloferula*) [6,19,20] and 2 *pmoA* lineages (i.e., USCα and MO3). Although our research on USCα (including RA14, JR1/Cluster 5 and MHP clade) is still insufficient, we already know a lot about its metabolism through studies of some MAGs [13,14,21] and an isolate strain [22]. In comparison, MO3 is currently the sole lineage of Beijerinckiaceae methanotrophs, whose metabolism is unknown. MO3 was initially enriched and detected in paddy soil and was later named as Cluster 4 [23,24]. MO3 has been detected in various soil environments [9] but is rarely found as a dominant methanotrophic group in environmental samples or methane-enriched samples [23,25,26].

In this study, we obtained a MO3-enriched culture by cultivating a paddy soil in the nitrogen-free M2 medium. Through metagenomic sequencing and assembly, we obtained a high-quality assembled genome of MO3 and further investigated its phylogeny and metabolic potential through the reconstruction of its central metabolism pathways.

## 2. Materials and Methods

### 2.1. Methanotrophic Enrichment

The paddy soil was collected from a typical subtropical agricultural region in China for rice–wheat rotation in Yangzhou City of Jiangsu province (119° 42′ 0″ E, 32° 35′ 5″ N). Soil cores (0–15 cm depth) were collected by a steel corer after rice harvest and stored at 4 °C until use. Soil characteristics were as follows: total organic carbon, 15 g kg^−1^; total nitrogen 1.59 g kg^−1^; total phosphorus 1.23 g kg^−1^, and pH 7.4. The soil was first incubated under 30% CH_4_ in 120 mL serum vials capped with butyl rubber stoppers to enrich methanotrophs, then 0.5 g enriched soil was transferred into 30 mL N-free M2 medium (no nitrate) [27] and incubated under 10% CH_4_ with shaking (150 rpm) for about a week. After-ward, 2 mL enriched medium was transferred into new N-free M2 medium and enriched for another three rounds. After each round, microbial cells in 2 mL enrichment culture were collected by centrifugation at 10,000 rpm. Soil and the cell pellets of each round were collected. Genomic DNA was extracted using the FastDNA spin kit for soil (MP Biomedicals, Santa Ana, CA, USA) in accordance with the manufacturer’s instructions and stored at –20 °C for amplicon sequencing.

### 2.2. MiSeq Sequencing and Analysis of 16S rRNA and pmoA Genes

The 16S rRNA and *pmoA* genes were amplified by primer pairs 515F/907R and A189f/mb661, respectively, as described previously to monitor the community changes of methanotrophs during multiple enrichment processes [28]. PCR products were purified by the MiniBEST DNA Fragment Purification Kit Ver.3.0 (TaKaRa) and quantified by the NanoDrop ND-1000 spectrophotometer and mixed at an equimolar ratio. The library was constructed using the TruSeq Nano DNA LT Sample Prep Kit Set A (24 samples), and sequencing was performed using the MiSeq Reagent Kit v3 (600 cycles).

Mothur (version 1.41.3) was used to process the raw sequence data [29]. For the 16S rRNA gene, reads with length of 370–380 nt were selected. Chimera detection and removal were conducted using the commands “chimera.vsearch” and “remove.seqs”, and the resulting high-quality reads were used for taxonomy classification by the “classify.seqs” command with a cutoff of 80% by using the “Wang” method. For *pmoA* gene, the commands “make.contigs” (deltaq = 5), and “trim.seqs” were used for merging of the paired-end reads, sample splitting, and preliminary quality control. These reads were then processed using the online version of the FunGene Pipeline [30] to check chimera by using the USEARCH 6.0 [31] and correct frameshifts by using the FramBot [32]. Finally, high-quality *pmoA* sequences were classified to known *pmoA* groups or lineages as previously described [10].

### 2.3. Metagenomic Sequencing, Assembly, and Binning

DNA from the fourth-round enrichment was used for metagenomic sequencing on the Illumina HiSeq 2500 platform with 2 × 150 bp paired-end cycles and resulted in 40 Gb of sequence data. Reads were assembled using the metaSPAdes v3.13.0 [33], MEGAHIT v1.2.9 [34], and IDBA-UD v1.1.3 [35] with the default parameters in the online service of KBase [36]. Metagenomic binning was performed on contigs longer than 1500 bp with the MetaBAT v2.12.1 [37] and MaxBin2 v2.2.4 [38] to obtain methanotroph MAGs [37]. The completeness and contamination of MAGs were assessed by the CheckM v1.0.17 [39]. GTDB-Tk v1.7.0 was used to make taxonomy classification of the obtained MAGs [40]. Gene features of methanotrophic MAGs were predicted by the prokka v1.14.5 [41] and prodigal [42]. The predicted amino-acid sequences of methanotrophic MAGs were annotated by the web tool BlastKOALA against the Kyoto Encyclopedia of Genes and Genomes (KEGG) database [41]. MAGs were also annotated by the RAST tool kit (RASTtk) in the online service of PATRIC [43,44,45]. The genomic map was generated using the CGView Server in accordance with the annotation results by BlastKOALA, RASTtk, and prokka [46].

### 2.4. Phylogeny Analysis

The full lengths of *pmoA*, *nifH*, and 16S rRNA genes extracted from methanotrophic MAGs were used to construct phylogenetic trees by using MEGA (version 6.06) to infer their phylogeny among known methanotrophs. A maximum-likelihood phylogenomic tree was also constructed with the FastTree v.2.1.10 [47] and visualized with ITOL after identifying and aligning a concatenated set of 120 marker proteins by using the GTDB-Tk v1.7.0 [48]. The genomic average nucleotide identity (gANI) and genomic average amino-acid identity (gAAI) values among methanotrophic MAGs and their related genomes were calculated by JSpeciesWS Online Service [49] and CompareM (https://github.com/dparks1134/CompareM accessed on 4 April 2022), respectively. Tools of Kostas lab were also used to calculate gANI and gAAI [50].

## 3. Results and Discussion

### 3.1. Succession of MOB in N-Free Medium

**Figure 1:**
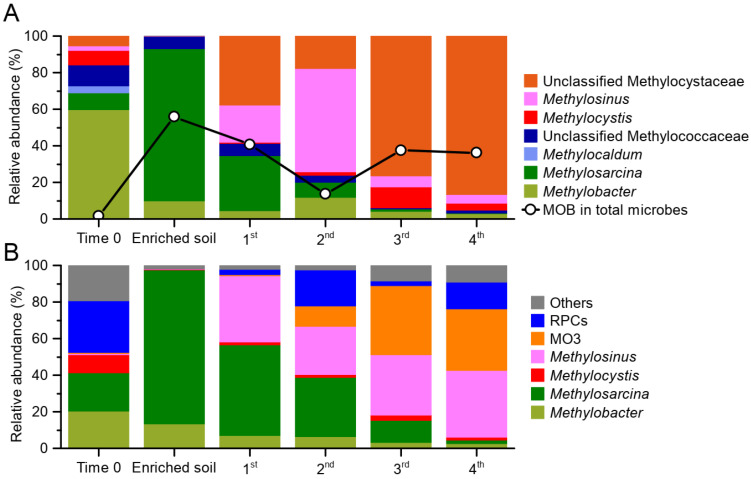
Changes in methanotrophic community compositions during enrichment in soil and nitrogen-free M2 medium. (**A**) Relative abundance of methanotrophs in total microorganisms and their community composition based on amplicon sequencing of partial 16S rRNA gene. (**B**) Changes in methanotrophic community composition based on amplicon sequencing of partial *pmoA* gene. 1st, 2nd, 3rd, and 4th mean the enrichment round in nitrogen-free M2 medium.

According to the amplicon-sequencing results of the 16S rRNA gene (Figure 1A), MOB accounts for about 1.6% of the total microorganisms in the original paddy soil. Their proportion in the soil reached 55.9% after the headspace CH_4_ was consumed, and after four additional rounds of enrichment in nitrogen-free liquid M2 medium, their proportion stabilized at about 36%. The dominant methanotroph in soil after enrichment is *Methylosarcina* (type I), which accounts for 84.3% of total methanotrophs. However, after four rounds of enrichment in N-free M2 medium, the dominant methanotrophs gradually changed into unclassified type II (Methylocystaceae), suggesting they are some novel taxa that have not been well-characterized. On the basis of the amplicon sequencing of the *pmoA* gene, we obtained similar results. After four rounds of enrichment, the dominant MOB is rapidly transformed from *Methylosarcina* to *Methylosinus* and MO3, of which the latter accounts for 33.4% of total MOB (Figure 1B). The actual proportion of MO3 may be much higher, because Beijerinckiaceae methanotrophs (type IIb) to which MO3 belongs generally have a single *pmoCAB* operon [22,51], whereas Methylocystaceae (type IIa) and other type I methanotrophs commonly have two *pmoCAB* operons in their genomes [52,53,54].

In most methanotroph-enrichment experiments using paddy soil, MO3 is rarely enriched [25,55,56]. We are not able to enrich it with NMS (nitrate mineral salts), nitrate-free NMS, and M2 media. The M2 medium is a fivefold dilution of M1 medium and is first designed for methanotrophs from freshwater wetlands and mildly acidic soils [27], and nitrate-free M2 medium is subsequently successfully used for enrichment and/or maintenance of multiple strains of Beijerinckiaceae methanotrophs, such as *Methylocella palustris* [57], *Methylocapsa acidiphila* [19], *Methylocella tundra* [58], and *Methylocapsa palsarum* [59]. Therefore, MO3 should have physiological characteristics similar to other Beijerinckiaceae methanotrophs, such as the ability to fix N_2_ and low-concentration inorganic salt requirements.

### 3.2. Reconstruction of MO3 MAGs

**Figure 2:**
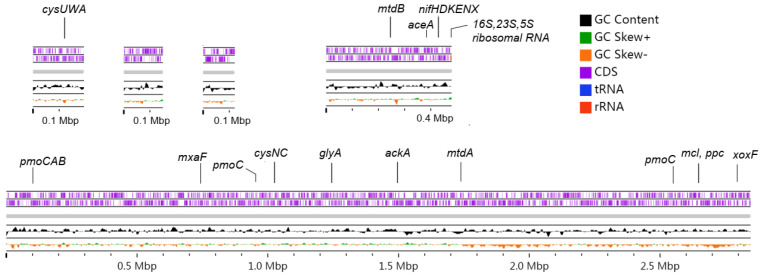
Main contigs of the reconstructed metagenome-assembled genome (MAG) of the *pmoA* lineage MO3 (MO3_YZ.1, Bin.033). The forward and reverse coding regions (CDS) of the five large contigs contained in this MAG were shown. Some genes encoding the key enzymes involved in carbon, nitrogen, and sulfur metabolism are marked in the outermost rings. Table S4 shows the full names of enzymes encoded by these genes. The other three contigs less than 30k in length were not shown.

DNA from the fourth round of enrichment is used for metagenomic sequencing. After reads assembly using three methods and contig binning using two methods, we obtained seven high-quality MOB MAGs (Table S1) with completeness > 92.5% and contamination < 2.63%. According to the classification results of GTDBkit, three MAGs belong to *Methylomagnum*, one MAG belongs to *Methylosinus*, and three MAGs belong to unknown Beijerinckiaceae. The gANI similarity of these three Beijerinckiaceae MAGs is over 99.6%, indicating that they belong to the same species, of which Bin.033 contains only eight contigs with completeness of 98.59% and contamination of 0.75% (Table 1). In addition, we detected a complete operon of ribosomal rRNA genes and complete operon of *pmoCAB* and *nifHDKENX* genes in Bin.033 (Figure 2, Tables S2–S4). Therefore, MAG Bin.033 was selected for subsequent analysis and labeled as MO3_YZ.1 (YZ indicates that this MAG originates from the soil sample collected from Yangzhou City).

### 3.3. Phylogeny of MO3

**Figure 3:**
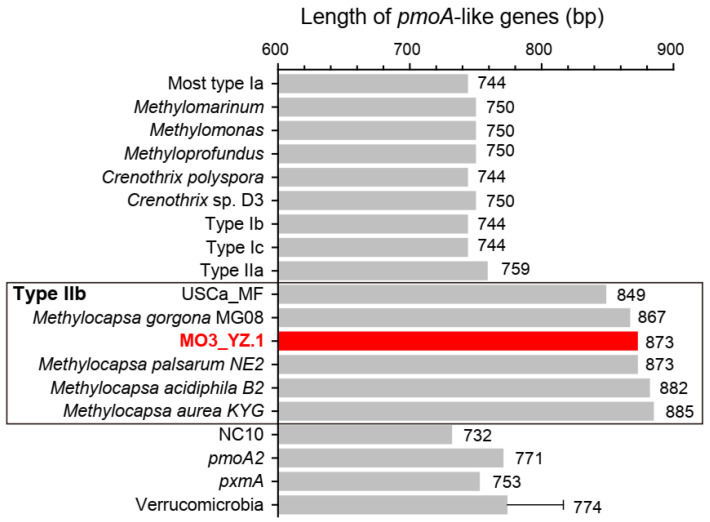
Length of *pmoA*-like genes in known methanotrophs. USCa_MF and *Methylocapsa* belong to family Beijerinckiaceae (type IIb). Length of Verrucomicrobia *pmoA* genes are shown as average value of multiple *pmoA* copies from several strains. The length of the *pmoA2* gene of *Methylocystis bryophila* S285 is 762 bp according to the current version (NZ_CP019948.1, 12-APR-2021) of its genome sequence. Table S5 shows more details.

MO3_YZ.1 has a *pmoA* gene length of 873 bp, is within the *pmoA* length range of type IIb (Beijerinckiaceae) methanotrophs, and is much longer than that of other methanotrophs (Figure 3, Table S5). The length of the *pmoA* gene can also serve as a taxonomic feature of methanotrophs. The *pmoA* genes of most type I methanotrophs are 744 bp in length, and only a few genera of type Ia such as *Methylomarinum*, *Methylomonas*, and *Methyloprofundus* have *pmoA* genes of 750 bp in length. Type IIa methanotrophs, including all species of *Methylocystis* and *Methylosinus*, have *pmoA* genes of 759 bp in length except *Methylocystis bryophila* S285 (762 bp). When the length of the *pmoA*-like sequence is 771 or 753 bp, it must be *pmoA2* or *pxmA* (Figure 3, Table S5). Therefore, in the future, when analyzing a methanotrophic MAG, the length of its *pmoA* gene sequence can help us make a preliminary judgment on the taxa to which it belongs.

**Figure 4:**
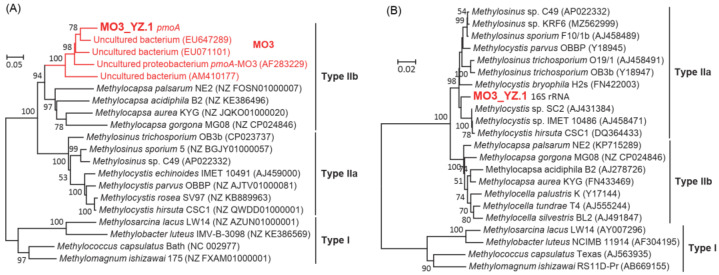
Phylogenetic relationship of MAG MO3_YZ.1 with its relatives on the basis of the full-length *pmoA* (**A**) and 16S rRNA (**B**) genes. Neighbor-joining trees were constructed using the MEGA 6.06 with 1000 replicates. Only bootstrap values higher than 50% are given at the branch nodes. Scale bars indicate 0.05 or 0.02 substitutions per nucleotide position.

**Figure 5:**
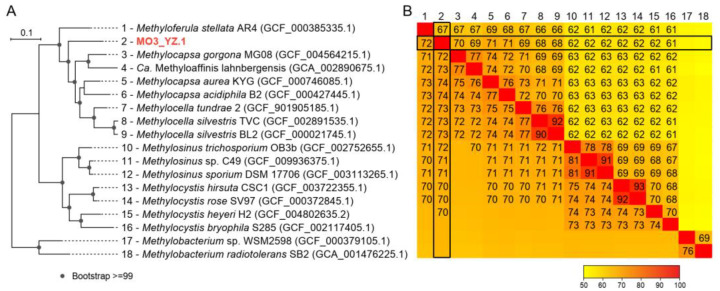
Phylogenetic relationship and pairwise genome-sequence similarity between MO3_YZ.1 and its relatives. (**A**) Genome tree showing the placement of MO3_YZ.1 within the Beijerinckiaceae methanotrophs. The maximum-likelihood phylogeny of representative reference genomes and MO3_YZ.1 was generated using a set of 120 concatenated marker proteins. Bootstrap values were calculated from 100 replicates. Scale bar equals 0.1 amino-acid substitutions per site. (**B**) Matrix of pairwise average nucleotide identity (gANI) and average amino-acid identity (gAAI) values between all these strains in the same order as indicated in (A). gANI was calculated by JSpeciesWS Online Service and presented in the lower-left triangle for values ≥70. gAAI was calculated by CompareM and presented in the upper-right triangle for values ≥60.

The phylogenetic analysis of the *pmoA* gene from MO3_YZ.1 confirms its affiliation to the MO3 lineage, which is closely related but distinct from *Methylocapsa*, the sole *pmoA*-containing genus of Beijerinckiaceae (Figure 4A). The phylogeny of *nifH* genes also shows a close relationship of MO3_YZ.1 to Beijerinckiaceae methanotrophs (Figure S1). However, when its 16S rRNA gene is used for phylogenetic tree construction, MO3_YZ.1 undoubtedly falls into the group of *Methylosysits*/*Methylosinus*, i.e., Methylocystaceae methanotrophs (type IIa, Figure 4B), and shows 98.5% of 16S rRNA sequence identity with *Methylosinus* sp. C49. The phylogenomic tree based on a concatenated set of 120 marker proteins confirms the placement of the MO3_YZ.1 within Beijerinckiaceae (Figure 5A). The maximum values of gANI and gAAI between MO3_YZ.1 and other known Beijerinckiaceae MOB genomes are 74% (by JSpeciesWS Online Service) and 71% (by CompareM), respectively (Figure 5B). When tools of Kostas lab are used for calculation, the maximum values of gANI and gAAI are 79% and 69%, respectively (Figure S2). Based on these similarity values, whether MO3 should be a new genus of Beijerinckiaceae or a new species of *Methylocapsa* cannot be concluded yet.

The phylogenies of 16S rRNA and *pmoA* genes from MO3_YZ.1 are not congruent as they affiliate to different families. Such case has not been reported within the known type II methanotrophs. The 16S rRNA genes often fail to assemble and bin due to their conserved and repetitive nature [60]. It should be treated with caution when the 16S rRNA gene of one MAG appears incongruent taxonomic classification with the taxonomic identity of this MAG [61]. Due to the conservation of the 16S rRNA gene, it is expected that the 16S rRNA gene of MO3_YZ.1 should be most related to *Methylocapsa*. Therefore, in this study, the assembled *Methylosinus*-like 16S rRNA gene in MO3_YZ.1 very likely does not belong to this MAG. It may be a fragment of contaminating sequence from a *Methylosinus* species due to the large proportion of *Methylosinus* in the enriched culture.

### 3.4. Methane-Oxidation Pathway of MO3

**Figure 6:**
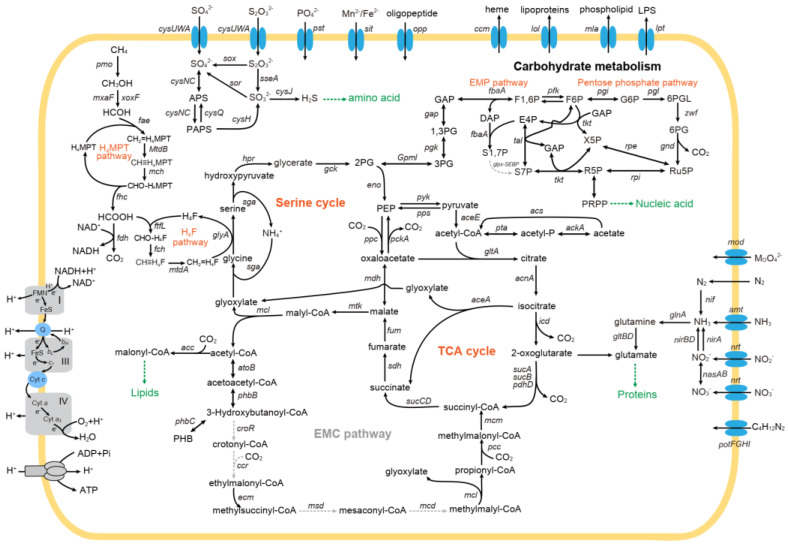
Metabolic reconstruction of MO3 based on the MAG MO3_YZ.1. Pathways are drawn on the basis of KEGG map files and KO assignments. Gray dashed arrows indicate the absence of these genes in this MAG. EMC, ethylmalonyl-CoA; EMP, Embden–Meyerhof–Parnas; LPS, lipopolysaccharide; PHB, poly-β-hydroxybutyrate; TCA, tricarboxylic acid.

We reconstructed the central metabolic pathways of MO3 on the basis of the gene-function annotation of MO3_YZ.1 (Figure 6). MO3_YZ.1 possesses a complete operon of *pmoCAB* genes coding the particulate methane monooxygenase and has two orphan *pmoC* genes (Figure 2). According to alignment of the deduced amino-acid sequences of *pmoA* genes, the amino acid of His38, Met42, Asp47, Asp49, and Glu100 for the tricopper cluster site is highly conserved in MO3_YZ.1 and other methanotrophs (Figure S3) as previously reported [62]. Like *Methylocapsa* species, other *pmoA*-like genes (*pxmA* and *pmoA2*) and the soluble methane monooxygenase coding genes are absent in MO3_YZ.1 [3]. We further identified coding genes of *mxaF*- and *xoxF*-type methanol dehydrogenase (MDH), which require calcium and lanthanide in their active center, respectively [63,64]. The *xoxF*-type MDH is a homodimer of the canonical *mxaF*-type MDH, and appears to be more widespread than the later. The *xoxF*-type MDH uses rare-earth elements as part of its catalytic center, and therefore the expression and activity of these two MDHs depends on the availability of rare-earth elements [63]. The *xoxF* gene of MO3 shows an amino-acid identity of 86.6% to that of *Methylocapsa aurea* (WP_036262132), and more than 79% to that of other Beijerinckiaceae methanotrophs, such as *Methylocapsa palsarum* NE2 [51], *Ca*. Methyloaffinis lahnbergensis [13], and *Methylocella silvestris* [65]. We also recovered a complete gene set of the tetrahydromethanopterin-dependent pathway (H_4_MPT pathway) for C1-carbon transfer during the oxidation of formaldehyde to formate, and *fdh* gene for the nonreversible formate dehydrogenase. MO3_YZ.1 catalyzes the final oxidation step of formate to CO_2_ and produces NADH, which can further drive the production of ATP through the respiratory chain. However, neither the coding genes of the carbon-monoxide dehydrogenase nor those of [NiFe] hydrogenase are identified in MO3_YZ.1, suggesting that MO3 cannot use CO and H_2_ as alternative energy sources as *Methylocapsa gorgona* MG08 [22].

### 3.5. Carbon Assimilation of MO3

We detected a complete gene set of the serine cycle for the assimilation of C1 from formate. Formate was condensed with tetrahydrofolate (H_4_F) to form formyl-H_4_F, which was transformed to methylene-H_4_F via the H_4_F pathway, and then methylene-H_4_F reacted with glycine to form serine (Figure 6). The regeneration of glyoxylate is a key pathway for the carbon assimilation of type II methanotrophs possessing serine cycle [66]. The coding gene (*aceA*) of the key enzyme (isocitrate lyase) of glyoxylate shunt and a complete gene set of the tricarboxylic acid (TCA) cycle in MO3_YZ.1 are observed, implying that the acetyl-CoA produced in the serine cycle can be subsequently oxidized to glyoxylate in assistance of some TCA cycle enzymes. This regeneration pathway of glyoxylate is common in type IIb but absent in type IIa methanotrophs, which use the ethylmalonyl-CoA (EMC) pathway to accomplish the same task [67]. Although many encoding genes of the EMC pathway-related enzymes are also detected in MO3_YZ.1, the encoding genes of four enzymes are absent (*croR* for 3-hydroxybutyryl-CoA dehydratase, *ccr* for crotonyl-CoA carboxylase/reductase, *msd* for 2-methylfumaryl-CoA hydratase and *mcd* for methenyltetrahydromethanopterin cyclohydrolase), indicating that MO3, like other Beijerinckiaceae methanotrophs, cannot regenerate glyoxylate through the EMC pathway. For MO3, the acetyl-CoA produced in the serine cycle can also be converted to poly-β-hydroxybutyrate (PHB, Figure 6). This carbon-storage polymer is also an endogenous source of reducing power [68], and may help MO3 adapt to environments with fluctuating substrate supplies [55,69].

As expected, the major carbon-assimilation pathway in type I methanotrophs, the RuMP pathway, is not retrieved in MO3_YZ.1 because the coding genes of the two key enzymes (*hps* for 3-hexulose-6-phosphate synthase and *phi* for 6-phospho-3-hexuloisomerase) of the RuMP pathway are absent in MO3_YZ.1. The coding gene of ribulose-bisphosphate carboxylase, the key enzyme of the Calvin–Benson–Bassham (CBB) cycle for CO_2_ fixation, is also absent in MO3_YZ.1. Thus, in this respect, MO3 is similar to *Methylocapsa gorgona* MG08 [22] and different to several other type IIb strains including *Methylocapsa acidiphila* [19], *Methylocapsa palsarum* NE2 [51], *Methylocella silvestris* BL2 [70], and *Methyloferula stellata* AR4 [71] which have a complete CBB cycle. MO3_YZ.1 encodes the Embden–Meyerhof–Parnas and pentose phosphate pathways for carbohydrate metabolism. In addition, MO3_YZ.1 carries all the necessary genes for enzymes involved in acetate metabolism, such as *acs* for acetate-CoA synthetase, *ackA* for acetate kinase, and *pta* for phosphotransacetylase (Figure 6). However, whether MO3 can grow using acetate as sole substrate like *Methylocapsa aurea* [72] is unknown because *Methylocapsa gorgona* MG08, which also carries these genes, cannot grow on acetate as the sole carbon source as expected [22]. An efficient membrane transporter for acetate (acetate permease ActP) may be necessary, but we currently know very little about this [52,73].

### 3.6. Nitrogen and Sulfur Metabolism of MO3

For nitrogen metabolism, MO3_YZ.1 possesses a complete *nifHDKENX* operon for molybdenum-containing nitrogenase like other type II methanotrophs [22,74] and genes for assimilatory nitrate reduction (*nasAB*, and *nirA*), dissimilatory nitrite reduction to ammonium (*nirBD*), ammonium transporter (*amt*), nitrate/nitrite transport protein (*nrt*), and putrescine transport-system protein (*potFGHI*) (Figure 6). The presence of these genes suggests that MO3 can utilize multiple types of nitrogen sources. As expected, genes encoding the denitrification pathway are missing in MO3_YZ.1 as many other aerobic methanotrophs [75]. For sulfur metabolism, MO3_YZ.1 possesses a series of genes in the sulfur-assimilation pathway (Figure 6). These genes include *cysUWA* (encodes sulfate/thiosulfate transport system permease/ATP-binding proteins); *cysNC*, *cysH*, and *cysJ* (encodes enzymes catalyze the subsequent sulfate-reduction steps to sulfide); and genes for sulfur-containing amino-acid production from sulfide (such as *cysE* and *cysK*) (Table S2). In addition, some genes encoding sulfur-oxidation enzymes, such as sulfite dehydrogenase (*sor*), thiosulfate/3-mercaptopyruvate sulfurtransferase (*sseA*) and S-sulfosulfanyl-L-cysteine sulfohydrolase (*sox*), are present in MO3_YZ.1. However, studies and discussions on the sulfur metabolism of aerobic methanotrophs are relatively few [14,76]. Whether sulfur metabolism is related to the carbon metabolism, energy acquisition, and environmental adaptability of methanotrophs remains to be investigated.

## 4. Conclusions

We enriched the uncultured Beijerinckiaceae methanotroph MO3 from paddy soil by using the nitrogen-free M2 medium and reconstructed a nearly complete genome of this lineage. Based on phylogenomic analysis, the closest relative of MO3 was *Methylocapsa*. In terms of the carbon-assimilation pathway, MO3 also exhibited similar characteristics to *Methylocapsa*. Its 16S rRNA gene was most related to *Methylosinus* rather than *Methylocapsa*, probably due to the typical misassembly of 16S rRNA gene from metagenomic data. MO3 encoded diverse metabolisms related to nitrogen, sulfur, and PHB, implying its ability to survive in a variety of stress environments such as low nitrogen availability.

## Figures and Tables

**Table 1:** Genome statistics of the obtained three metagenome-assembled genomes (MAGs) of lineage MO3.

Parameter	Bin.033	Bin.053	Bin.006
Assembly method	MetaSPAdes	IDBA	MetaSPAdes
Binning method	MetaBAT2	MetaBAT2	MaxBin2
Size (M)	3.83	3.67	3.93
Completeness (%)	98.95	98.28	98.59
Contamination (%)	0.75	0.75	2.63
Taxa	Beijerinckiaceae	Beijerinckiaceae	Beijerinckiaceae
GC Content (%)	65.6	65.7	65.5
Number of Contigs	8	72	35
Number of genes	3442	3325	3511
Number of tRNAs	50	46	49
rRNA operon	5S (1), 16S (1), 23S (1)	5S (1)	5S (1), 16S (1), 23S (1)
*pmo* operon	1	1	1
*nif* operon	1	1	1

## Data Availability

The raw amplicon sequence datasets for 16S rRNA and *pmoA* genes have been deposited at the NCBI Sequence Read Archive (SRA) under BioProject number PRJNA480368. The MO3 draft genome MO3_YZ.1 has been deposited in GenBank under accession number JALJOM000000000.

## Supplementary Materials

The following supporting information can be downloaded at: https://www.mdpi.com/article/10.3390/microorganisms10050955/s1, Figure S1: Neighbor-joining phylogenetic tree of the *nifH* gene from MO3_YZ.1. The tree based on 290 amino-acid positions is constructed using MEGA software (version 6.06) and evaluated with 1000 bootstraps. Bootstrap values higher than 50% are given at the branch nodes. Scale bar indicates 2% amino-acid sequence divergence; Figure S2: Matrix of pairwise genomic average nucleotide identity (gANI) and genomic average amino-acid identity (gAAI) values of MO3_YZ.1 and its relatives. The genomes’ sequences were ordered as in Figure 4. The gANI was presented in the lower-left triangle and the gAAI was presented in the upper-right triangle. Bothe of gANI and gAAI in this figure were calculated by tools of Kostas lab. Figure S3. Alignments of amino-acid sequences of PmoA subunit from methanotrophs. The amino acids that form the tricopper cluster site are shown in blue. Table S1: Genome statistics of the obtained metagenome-assembled genomes (MAGs) affiliated to methanotrophs; Table S2: Gene features of Bin.033 predicted by prokka v1.14.5; Table S3: Gene features of Bin.033 annotated by RAST tool kit; Table S4: Gene functions of Bin.033 annotated by BlastKOALA through against the Kyoto Encyclopedia of Genes and Genomes database; Table S5: Length of *pmoA*-like genes in genomes of currently known aerobic methanotrophs.

## References

[1] Hanson R., Hanson T. (1996). Methanotrophic bacteria. Microbiol. Rev..

[2] Trotsenko Y.A., Murrell J.C. (2008). Metabolic aspects of aerobic obligate methanotrophy. Adv. Appl. Microbiol..

[3] Kalyuzhnaya M.G., Gomez O.A., Murrell J.C. (2019). The methane-oxidizing bacteria (methanotrophs). Taxonomy, genomics and ecophysiology of hydrocarbon-degrading microbes.

[4] Knief C. (2019). Diversity of methane-cycling microorganisms in soils and their relation to oxygen. Curr. Issues Mol. Biol..

[5] Pol A., Heijmans K., Harhangi H.R., Tedesco D., Jetten M.S.M., Op den Camp H.J.M. (2007). Methanotrophy below pH 1 by a new Verrucomicrobia species. Nature.

[6] Vorobev A.V., Baani M., Doronina N.V., Brady A.L., Liesack W., Dunfield P.F., Dedysh S.N. (2011). *Methyloferula stellata* gen. nov., sp. nov., an acidophilic, obligately methanotrophic bacterium that possesses only a soluble methane monooxygenase. Int. J. Syst. Evol. Microbiol..

[7] Theisen A.R., Ali M.H., Radajewski S., Dumont M.G., Dunfield P.F., McDonald I.R., Dedysh S.N., Miguez C.B., Murrell J.C. (2005). Regulation of methane oxidation in the facultative methanotroph *Methylocella silvestris* BL2. Mol. Microbiol..

[8] Vekeman B., Kerckhof F.-M., Cremers G., de Vos P., Vandamme P., Boon N., Op den Camp H.J.M., Heylen K. (2016). New *Methyloceanibacter* diversity from North Sea sediments includes methanotroph containing solely the soluble methane monooxygenase. Environ. Microbiol..

[9] Knief C. (2015). Diversity and habitat preferences of cultivated and uncultivated aerobic methanotrophic bacteria evaluated based on *pmoA* as molecular marker. Front. Microbiol..

[10] Dumont M.G., Lüke C., Deng Y., Frenzel P. (2014). Classification of *pmoA* amplicon pyrosequences using BLAST and the lowest common ancestor method in MEGAN. Front. Microbiol..

[11] Zheng Y., Chistoserdova L. (2019). Multi-omics understanding of methanotrophs. Methanotrophs.

[12] Edwards C.R., Onstott T.C., Miller J.M., Wiggins J.B., Wang W., Lee C.K., Cary S.C., Pointing S.B., Lau M.C.Y. (2017). Draft genome sequence of uncultured upland soil cluster gammaproteobacteria gives molecular insights into high-affinity methanotrophy. Genome Announc..

[13] Pratscher J., Vollmers J., Wiegand S., Dumont M.G., Kaster A.-K. (2018). Unravelling the identity, metabolic potential and global biogeography of the atmospheric methane-oxidizing upland soil cluster α. Environ. Microbiol..

[14] Singleton C.M., McCalley C.K., Woodcroft B.J., Boyd J.A., Evans P.N., Hodgkins S.B., Chanton J.P., Frolking S., Crill P.M., Saleska S.R. (2018). Methanotrophy across a natural permafrost thaw environment. ISME J..

[15] Jung G.-Y., Rhee S.-K., Han Y.-S., Kim S.-J. (2020). Genomic and physiological properties of a facultative methane-oxidizing bacterial strain of *Methylocystis* sp. from a wetland. Microorganisms.

[16] Smith G.J., Angle J.C., Solden L.M., Borton M.A., Morin T.H., Daly R.A., Johnston M.D., Stefanik K.C., Wolfe R., Gil B. (2018). Members of the genus *Methylobacter* are inferred to account for the majority of aerobic methane oxidation in oxic soils from a freshwater wetland. mBio.

[17] Chen L.-X., Anantharaman K., Shaiber A., Eren A.M., Banfield J.F. (2020). Accurate and complete genomes from metagenomes. Genome Res..

[18] Quince C., Walker A.W., Simpson J.T., Loman N.J., Segata N. (2017). Shotgun metagenomics, from sampling to analysis. Nat. Biotechnol..

[19] Dedysh S.N., Khmelenina V.N., Suzina N.E., Trotsenko Y.A., Semrau J.D., Liesack W., Tiedje J.M. (2002). *Methylocapsa acidiphila* gen. nov., sp. nov., a novel methane-oxidizing and dinitrogen-fixing acidophilic bacterium from Sphagnum bog. Int. J. Syst. Evol. Microbiol..

[20] Dunfield P.F., Khmelenina V.N., Suzina N.E., Trotsenko Y.A., Dedysh S.N. (2003). *Methylocella silvestris* sp. nov., a novel methanotroph isolated from an acidic forest cambisol. Int. J. Syst. Evol. Microbiol..

[21] Rusley C., Onstott T.C., Vishnivetskaya T.A., Layton A., Chauhan A., Pfiffner S.M., Whyte L.G., Lau M.C.Y. (2019). Metagenome-assembled genome of USCα AHI, a potential high-affinity methanotroph from axel heiberg island, Canadian high arctic. Microbiol. Resour. Ann..

[22] Tveit A.T., Hestnes A.G., Robinson S.L., Schintlmeister A., Dedysh S.N., Jehmlich N., von Bergen M., Herbold C., Wagner M., Richter A. (2019). Widespread soil bacterium that oxidizes atmospheric methane. Proc. Natl. Acad. Sci. USA.

[23] Henckel T., Roslev P., Conrad R. (2000). Effects of O_2_ and CH_4_ on presence and activity of the indigenous methanotrophic community in rice field soil. Environ. Microbiol..

[24] Knief C., Kolb S., Bodelier P.L., Lipski A., Dunfield P.F. (2006). The active methanotrophic community in hydromorphic soils changes in response to changing methane concentration. Environ. Microbiol..

[25] Zhao J., Cai Y., Jia Z. (2020). The pH-based ecological coherence of active canonical methanotrophs in paddy soils. Biogeosciences.

[26] Lüke C., Krause S., Cavigiolo S., Greppi D., Lupotto E., Frenzel P. (2010). Biogeography of wetland rice methanotrophs. Environ. Microbiol..

[27] Dedysh S.N., Panikov N.S., Tiedje J.M. (1998). Acidophilic methanotrophic communities from Sphagnum peat bogs. Appl. Environ. Microbiol..

[28] Cai Y., Zhou X., Shi L., Jia Z. (2020). Atmospheric methane oxidizers are dominated by upland soil cluster alpha in 20 forest soils of China. Microb. Ecol..

[29] Schloss P.D., Westcott S.L., Ryabin T., Hall J.R., Hartmann M., Hollister E.B., Lesniewski R.A., Oakley B.B., Parks D.H., Robinson C.J. (2009). Introducing mothur: Open-source, platform-independent, community-supported software for describing and comparing microbial communities. Appl. Environ. Microbiol..

[30] Fish J.A., Chai B.L., Wang Q., Sun Y.N., Brown C.T., Tiedje J.M., Cole J.R. (2013). FunGene: The functional gene pipeline and repository. Front. Microbiol..

[31] Edgar R.C., Haas B.J., Clemente J.C., Quince C., Knight R. (2011). UCHIME improves sensitivity and speed of chimera detection. Bioinformatics.

[32] Wang Q., Quensen J.F., Fish J.A., Lee T.K., Sun Y., Tiedje J.M., Cole J.R. (2013). Ecological patterns of *nifH* genes in four terrestrial climatic zones explored with targeted metagenomics using frameBot, a new informatics tool. mBio.

[33] Nurk S., Meleshko D., Korobeynikov A., Pevzner P.A. (2017). metaSPAdes: A new versatile metagenomic assembler. Genome Res..

[34] Li D., Liu C.-M., Luo R., Sadakane K., Lam T.-W. (2015). MEGAHIT: An ultra-fast single-node solution for large and complex metagenomics assembly via succinct de Bruijn graph. Bioinformatics.

[35] Peng Y., Leung H.C., Yiu S.-M., Chin F.Y. (2012). IDBA-UD: A de novo assembler for single-cell and metagenomic sequencing data with highly uneven depth. Bioinformatics.

[36] Arkin A.P., Cottingham R.W., Henry C.S., Harris N.L., Stevens R.L., Maslov S., Dehal P., Ware D., Perez F., Canon S. (2018). KBase: The United States department of energy systems biology knowledgebase. Nat. Biotechnol..

[37] Kang D.D., Froula J., Egan R., Wang Z. (2015). MetaBAT, an efficient tool for accurately reconstructing single genomes from complex microbial communities. PeerJ.

[38] Wu Y.-W., Simmons B.A., Singer S.W. (2016). MaxBin 2.0: An automated binning algorithm to recover genomes from multiple metagenomic datasets. Bioinformatics.

[39] Parks D.H., Imelfort M., Skennerton C.T., Hugenholtz P., Tyson G.W. (2015). CheckM: Assessing the quality of microbial genomes recovered from isolates, single cells, and metagenomes. Genome Res..

[40] Parks D.H., Chuvochina M., Waite D.W., Rinke C., Skarshewski A., Chaumeil P.-A., Hugenholtz P. (2018). A standardized bacterial taxonomy based on genome phylogeny substantially revises the tree of life. Nat. Biotechnol..

[41] Seemann T. (2014). Prokka: Rapid prokaryotic genome annotation. Bioinformatics.

[42] Hyatt D., Chen G.-L., Locascio P.F., Land M.L., Larimer F.W., Hauser L.J. (2010). Prodigal: Prokaryotic gene recognition and translation initiation site identification. BMC Bioinformatics.

[43] Kanehisa M., Sato Y., Morishima K. (2016). BlastKOALA and GhostKOALA: KEGG tools for functional characterization of genome and metagenome sequences. J. Mol. Biol..

[44] Wattam A.R., Davis J.J., Assaf R., Boisvert S., Brettin T., Bun C., Conrad N., Dietrich E.M., Disz T., Gabbard J.L. (2017). Improvements to PATRIC, the all-bacterial bioinformatics database and analysis resource center. Nucleic Acids Res..

[45] Brettin T., Davis J.J., Disz T., Edwards R.A., Gerdes S., Olsen G.J., Olson R., Overbeek R., Parrello B., Pusch G.D. (2015). RASTtk: A modular and extensible implementation of the RAST algorithm for building custom annotation pipelines and annotating batches of genomes. Sci. Rep..

[46] Grant J.R., Stothard P. (2008). The CGView Server: A comparative genomics tool for circular genomes. Nucleic Acids Res..

[47] Price M.N., Dehal P.S., Arkin A.P. (2010). FastTree 2–approximately maximum-likelihood trees for large alignments. PLoS ONE.

[48] Chaumeil P.-A., Mussig A.J., Hugenholtz P., Parks D.H. (2020). GTDB-Tk: A toolkit to classify genomes with the Genome Taxonomy Database. Bioinformatics.

[49] Richter M., Rossello-Mora R., Gloeckner F.O., Peplies J. (2016). JSpeciesWS: A web server for prokaryotic species circumscription based on pairwise genome comparison. Bioinformatics.

[50] Rodriguez-R L.M., Konstantinidis K.T. (2016). The enveomics collection: A toolbox for specialized analyses of microbial genomes and metagenomes. PeerJ Prepr..

[51] Miroshnikov K.K., Didriksen A., Naumoff D.G., Huntemann M., Clum A., Pillay M., Palaniappan K., Varghese N., Mikhailova N., Mukherjee S. (2017). Draft genome sequence of *Methylocapsa palsarum* NE2T, an obligate methanotroph from subarctic soil. Genome Announc..

[52] Han D., Dedysh S.N., Liesack W. (2018). Unusual genomic traits suggest *Methylocystis bryophila* S285 to be well adapted for life in peatlands. Genome Biol. Evol..

[53] Stein L.Y., Yoon S., Semrau J.D., DiSpirito A.A., Crombie A., Murrell J.C., Vuilleumier S., Kalyuzhnaya M.G., den Camp H., Bringel F.O. (2010). Genome sequence of the obligate methanotroph *Methylosinus trichosporium* strain OB3b. J. Bacteriol..

[54] Stolyar S., Costello A.M., Peeples T.L., Lidstrom M.E. (1999). Role of multiple gene copies in particulate methane monooxygenase activity in the methane-oxidizing bacterium *Methylococcus capsulatus* Bath. Microbiology.

[55] Cai Y., Zheng Y., Bodelier P.L.E., Conrad R., Jia Z. (2016). Conventional methanotrophs are responsible for atmospheric methane oxidation in paddy soils. Nat. Commun..

[56] Shiau Y.-J., Cai Y., Jia Z., Chen C.-L., Chiu C.-Y. (2018). Phylogenetically distinct methanotrophs modulate methane oxidation in rice paddies across Taiwan. Soil Biol. Biochem..

[57] Dedysh S.N., Liesack W., Khmelenina V.N., Suzina N.E., Trotsenko Y.A., Semrau J.D., Bares A.M., Panikov N.S., Tiedje J.M. (2000). *Methylocella palustris* gen. nov., sp. nov., a new methane-oxidizing acidophilic bacterium from peat bogs, representing a novel subtype of serine-pathway methanotrophs. Int. J. Syst. Evol. Microbiol..

[58] Dedysh S.N., Berestovskaya Y.Y., Vasylieva L.V., Belova S.E., Khmelenina V.N., Suzina N.E., Trotsenko Y.A., Liesack W., Zavarzin G.A. (2004). *Methylocella tundrae* sp. nov., a novel methanotrophic bacterium from acidic tundra peatlands. Int. J. Syst. Evol. Microbiol..

[59] Dedysh S.N., Didriksen A., Danilova O.V., Belova S.E., Liebner S., Svenning M.M. (2015). *Methylocapsa palsarum* sp. nov., a methanotroph isolated from a subArctic discontinuous permafrost ecosystem. Int. J. Syst. Evol. Microbiol..

[60] Yuan C., Lei J., Cole J., Sun Y. (2015). Reconstructing 16S rRNA genes in metagenomic data. Bioinformatics.

[61] Parks D.H., Rinke C., Chuvochina M., Chaumeil P.-A., Woodcroft B.J., Evans P.N., Hugenholtz P., Tyson G.W. (2017). Recovery of nearly 8,000 metagenome-assembled genomes substantially expands the tree of life. Nat. Microbial..

[62] Wang V.C.-C., Maji S., Chen P.P.-Y., Lee H.K., Yu S.S.-F., Chan S.I. (2017). Alkane oxidation: Methane monooxygenases, related enzymes, and their biomimetics. Chem. Rev..

[63] Chistoserdova L. (2016). Lanthanides: New life metals?. World J. Microbiol. Biotechnol..

[64] Keltjens J.T., Pol A., Reimann J., Op den Camp H.J. (2014). PQQ-dependent methanol dehydrogenases: Rare-earth elements make a difference. Appl. Microbiol. Biotechnol..

[65] Wang J., Geng K., Haque M.F.U., Crombie A., Street L.E., Wookey P.A., Ma K., Murrell J.C., Pratscher J. (2018). Draft genome sequence of *Methylocella silvestris* TVC, a facultative methanotroph isolated from permafrost. Genome Announc..

[66] Anthony C. (2011). How half a century of research was required to understand bacterial growth on C1 and C2 compounds; the story of the serine cycle and the ethylmalonyl-CoA pathway. Sci. Prog..

[67] Kalyuzhnaya M.G., Eckert C.A., Trinh C.T. (2016). Chapter 13 - Methane Biocatalysis: Selecting the Right Microbe. Biotechnology for Biofuel Production and Optimization.

[68] Henrysson T., McCarty P.L. (1993). Influence of the endogenous storage lipid poly-β-hydroxybutyrate on the reducing power availability during cometabolism of trichloroethylene and naphthalene by resting methanotrophic mixed cultures. Appl. Environ. Microbiol..

[69] Sipkema E.M., de Koning W., Ganzeveld K.J., Janssen D.B., Beenackers A. (2000). NADH-regulated metabolic model for growth of *Methylosinus trichosporium* OB3b. Model presentation, parameter estimation, and model validation. Biotechnol. Prog..

[70] Chen Y., Crombie A., Rahman M.T., Dedysh S.N., Liesack W., Stott M.B., Alam M., Theisen A.R., Murrell J.C., Dunfield P.F. (2010). Complete genome sequence of the aerobic facultative methanotroph *Methylocella silvestris* BL2. J. Bacteriol..

[71] Dedysh S.N., Naumoff D.G., Vorobev A.V., Kyrpides N., Woyke T., Shapiro N., Crombie A.T., Murrell J.C., Kalyuzhnaya M.G., Smirnova A.V. (2015). Draft genome sequence of *Methyloferula stellata* AR4, an obligate methanotroph possessing only a soluble methane monooxygenase. Genome Announc..

[72] Dunfield P.F., Belova S.E., Vorob’ev A.V., Cornish S.L., Dedysh S.N. (2010). *Methylocapsa aurea* sp. nov., a facultative methanotroph possessing a particulate methane monooxygenase, and emended description of the genus *Methylocapsa*. Int. J. Syst. Evol. Microbiol..

[73] Haque M.F.U., Xu H.-J., Murrell J.C., Crombie A. (2020). Facultative methanotrophs–diversity, genetics, molecular ecology and biotechnological potential: A mini-review. Microbiology.

[74] Dedysh S.N., Ricke P., Liesack W. (2004). NifH and NifD phylogenies: An evolutionary basis for understanding nitrogen fixation capabilities of methanotrophic bacteria. Microbiology.

[75] Stein L.Y., Klotz M.G. (2011). Nitrifying and denitrifying pathways of methanotrophic bacteria. Biochem. Soc. Trans..

[76] Ghashghavi M., Belova S.E., Bodelier P.L., Dedysh S.N., Kox M.A., Speth D.R., Frenzel P., Jetten M.S., Lücker S., Lüke C. (2019). *Methylotetracoccus oryzae* strain C50C1 is a novel type Ib gammaproteobacterial methanotroph adapted to freshwater environments. mSphere.

