# Supplementary material for: Phylogeny and Metabolic Potential of the Methanotrophic Lineage MO3 in Beijerinckiaceae from the Paddy Soil through Metagenome-Assembled Genome Reconstruction
Source: Microorganisms. 2022 May 1;10(5):955. doi: 10.3390/microorganisms10050955 (PMC9145241; doi:10.3390/microorganisms10050955)
Supplement: Supplementary file 1 [file microorganisms-10-00955-s001.zip › Cai YF-et al-2022.4.18-SI.pdf]

Table S1. Genome statistics of the obtained metagenome-assembled genomes (MAGs) affiliated to methanotrophs.

**MAGs**	**Assemble method**	**Binning method**	**Comple teness**	**Contamin ation**	**Classification by GTDBkit**
Bin.025	IDBA	MetaBAT2	92.54	2.28	d__Bacteria;p__Proteobacteria;c__Gammaproteobacteria;o__Methylococcales;f__Methylococcaceae;g__Methylomagnum;s__
Bin.053	IDBA	MetaBAT2	98.28	0.75	d__Bacteria;p__Proteobacteria;c__Alphaproteobacteria;o__Rhizobiales;f__Beijerinckiaceae;g__;s__
Bin.078	MEGAHIT	MetaBAT2	95.31	2.28	d__Bacteria;p__Proteobacteria;c__Gammaproteobacteria;o__Methylococcales;f__Methylococcaceae;g__Methylomagnum;s__
Bin.006	MetaSPAdes	MaxBin2	98.59	2.63	d__Bacteria;p__Proteobacteria;c__Alphaproteobacteria;o__Rhizobiales;f__Beijerinckiaceae;g__;s__
Bin.016	MetaSPAdes	MetaBAT2	93.27	0.63	d__Bacteria;p__Proteobacteria;c__Alphaproteobacteria;o__Rhizobiales;f__Beijerinckiaceae;g__Methylosinus;s__Methylosinus sporium
Bin.033	MetaSPAdes	MetaBAT2	98.59	0.75	d__Bacteria;p__Proteobacteria;c__Alphaproteobacteria;o__Rhizobiales;f__Beijerinckiaceae;g__;s__
Bin.035	MetaSPAdes	MetaBAT2	98.79	2.45	d__Bacteria;p__Proteobacteria;c__Gammaproteobacteria;o__Methylococcales;f__Methylococcaceae;g__Methylomagnum;s__

Table S2. Gene features of Bin.033 predicted by prokka v1.14.5. This table has been provided as a separate Excel file.

Table S3. Gene features of Bin.033 annotated by RAST tool kit. This table has been provided as a separate Excel file.

Table S4. Gene functions of Bin.033 annotated by BlastKOALA through against the Kyoto Encyclopedia of Genes and Genomes database. This table has been provided as a separate Excel file.

Table S5. Length of *pmoA*-like genes in genomes of currently known aerobic methanotrophs. This table has been provided as a separate Excel file.

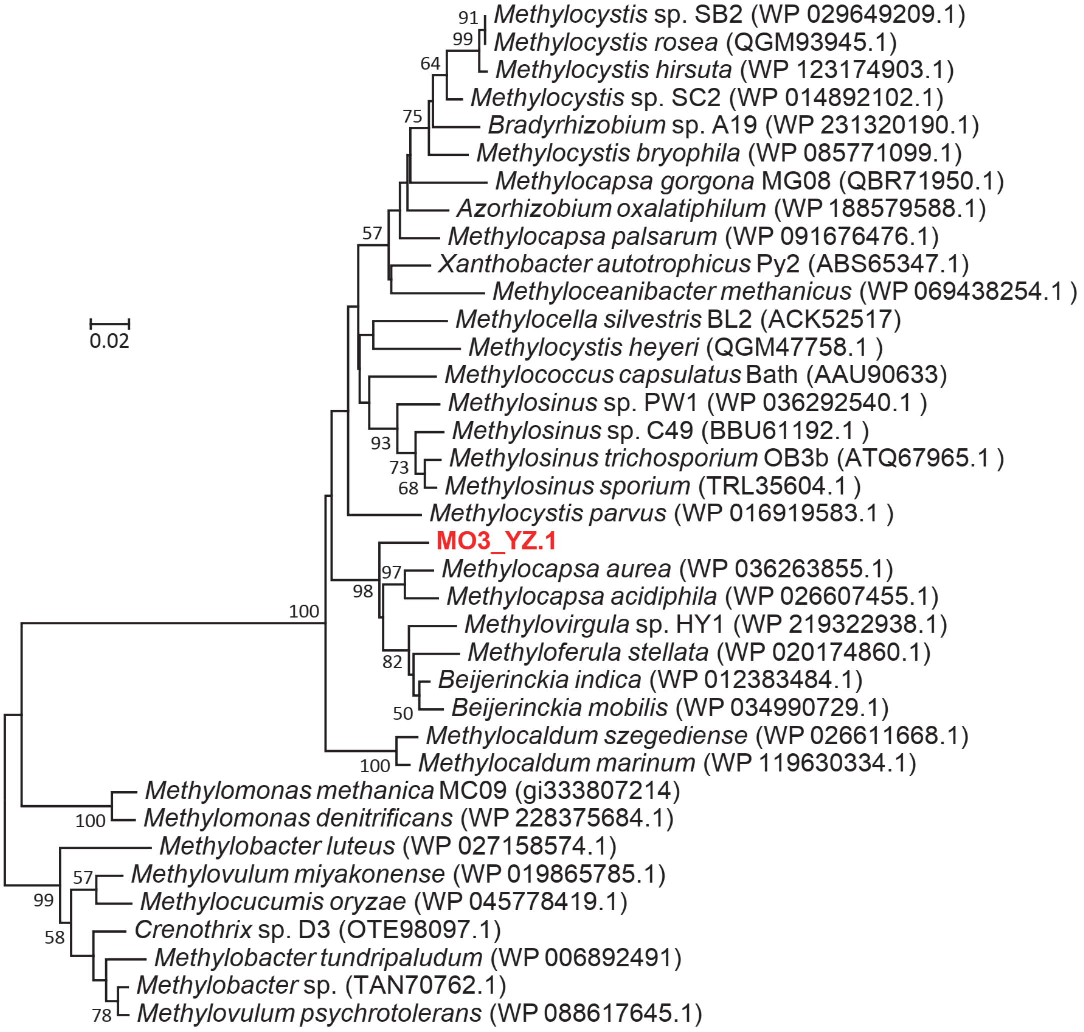

Figure S1. Neighbour-joining phylogenetic tree of the *nifH* gene from MO3_YZ.1. The tree based on 290 amino acid positions is constructed using MEGA software (version 6.06) and evaluated with 1000 bootstraps. Bootstrap values higher than 50% are given at the branch nodes. Scale bar indicates 2% amino acid sequence divergence.

Average nucleotide identity (gANI)

	1	2	3	4	5	6	7	8	9	10	11	12	13	14	15	16	17	18
1 - *Methyloferula stellata* AR4 (GCF_000385335.1)	100	63	64	61	65	65	64	63	63	57	57	58	57	57	57	56	54	54
2 - **MO3_YZ.1**	77	100	67	64	69	68	64	64	64	58	58	58	57	58	57	56	54	54
3 - *Methylocapsa gorgona* MG08 (GCF_004564215.1)	77	77	100	72	72	70	68	66	66	58	59	59	58	59	58	58	54	54
4 - *Ca.* Methyloaffinis lahnbergensis (GCA_002890675.1)	77	77	79	100	69	67	65	63	63	56	57	56	56	56	56	55	52	52
5 - *Methylocapsa aurea* KYG (GCF_000746085.1)	78	78	78	79	100	74	70	68	69	59	59	59	58	59	58	57	55	54
6 - *Methylocapsa acidiphila* B2 (GCF_000427445.1)	78	79	78	78	80	100	69	67	67	59	59	60	59	58	58	58	54	54
7 - *Methylocella tundrae* 2 (GCF_901905185.1)	77	78	77	77	79	79	100	75	75	58	58	58	58	58	58	57	54	54
8 - *Methylocella silvestris* TVC (GCF_002891535.1)	78	78	77	77	78	78	80	100	92	58	58	58	57	58	57	57	54	54
9 - *Methylocella silvestris* BL2 (GCF_000021745.1)	78	78	77	77	78	78	80	91	100	58	58	58	57	58	57	56	54	54
10 - *Methylosinus trichosporium* OB3b (GCF_002752655.1)	77	77	75	75	77	77	77	77	77	100	76	76	65	65	65	64	53	53
11 - *Methylosinus* sp. C49 (GCF_009936375.1)	77	77	75	75	76	77	76	77	77	84	100	90	66	66	66	64	53	53
12 - *Methylosinus sporium* DSM 17706 (GCF_003113265.1)	77	77	76	75	77	77	76	77	77	84	91	100	66	66	66	65	53	53
13 - *Methylocystis hirsuta* CSC1 (GCF_003722355.1)	77	77	75	75	77	76	76	76	77	79	79	78	100	92	66	65	52	52
14 - *Methylocystis rose* SV97 (GCF_000372845.1)	77	77	76	75	76	76	77	77	77	79	78	78	92	100	66	65	54	53
15 - *Methylocystis heyeri* H2 (GCF_004802635.2)	76	77	75	75	76	76	76	76	76	79	79	79	79	79	100	68	52	52
16 - *Methylocystis bryophila* S285 (GCF_002117405.1)	76	76	75	75	76	76	76	77	76	79	78	78	78	78	79	100	52	52
17 - *Methylobacterium* sp. WSM2598 (GCF_000379105.1)	75	76	74	74	75	76	76	76	76	77	76	77	75	76	76	76	100	66
18 - *Methylobacterium radiotolerans* SB2 (GCA_001476225.1)	75	76	74	73	75	76	75	76	76	76	76	76	76	76	76	76	80	100

Average amino acid identity (gAAI)

Figure S2. Matrix of pairwise genomic average nucleotide identity (gANI) and genomic average amino acid identity (gAAI) values of MO3_YZ.1 and its relatives. The genomes sequences were ordered as in Figure 4. The gANI was presented in the lower left triangle and the gAAI was presented in the upper right triangle. Bothe of gANI and gAAI in this figure were calculated by tools of Kostas lab.

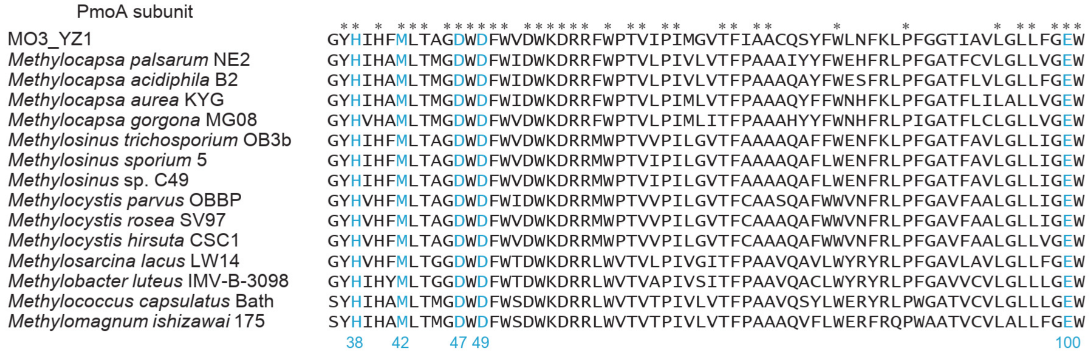

Figure S3. Alignments of amino acid sequences of PmoA subunit from methanotrophs. The amino acids that form the tricopper cluster site are shown in blue.
